# Neurocomputational model of compulsivity: deviating from an uncertain goal-directed system

**DOI:** 10.1093/brain/awae102

**Published:** 2024-04-08

**Authors:** Taekwan Kim, Sang Wan Lee, Silvia Kyungjin Lho, Sun-Young Moon, Minah Kim, Jun Soo Kwon

**Affiliations:** Department of Brain and Cognitive Sciences, Seoul National University College of Natural Sciences, Seoul 08826, Republic of Korea; Department of Bio and Brain Engineering, Korea Advanced Institute of Science and Technology, Daejeon 34141, Republic of Korea; Center for Neuroscience-inspired Artificial Intelligence, Korea Advanced Institute of Science and Technology, Daejeon 34141, Republic of Korea; Department of Bio and Brain Engineering, Korea Advanced Institute of Science and Technology, Daejeon 34141, Republic of Korea; Center for Neuroscience-inspired Artificial Intelligence, Korea Advanced Institute of Science and Technology, Daejeon 34141, Republic of Korea; Kim Jaechul Graduate School of AI, Korea Advanced Institute of Science and Technology, Daejeon 34141, Republic of Korea; Department of Neuropsychiatry, Seoul National University Hospital, Seoul 03080, Republic of Korea; Department of Neuropsychiatry, Seoul National University Hospital, Seoul 03080, Republic of Korea; Department of Neuropsychiatry, Seoul National University Hospital, Seoul 03080, Republic of Korea; Department of Psychiatry, Seoul National University College of Medicine, Seoul 03080, Republic of Korea; Department of Brain and Cognitive Sciences, Seoul National University College of Natural Sciences, Seoul 08826, Republic of Korea; Department of Neuropsychiatry, Seoul National University Hospital, Seoul 03080, Republic of Korea; Department of Psychiatry, Seoul National University College of Medicine, Seoul 03080, Republic of Korea

**Keywords:** obsessive-compulsive disorder, compulsivity, arbitration, goal-directed behaviour, neurocomputational model

## Abstract

Despite a theory that an imbalance in goal-directed versus habitual systems serve as building blocks of compulsions, research has yet to delineate how this occurs during arbitration between the two systems in obsessive-compulsive disorder. Inspired by a brain model in which the inferior frontal cortex selectively gates the putamen to guide goal-directed or habitual actions, this study aimed to examine whether disruptions in the arbitration process via the fronto-striatal circuit would underlie imbalanced decision-making and compulsions in patients.

Thirty patients with obsessive-compulsive disorder [mean (standard deviation) age = 26.93 (6.23) years, 12 females (40%)] and 30 healthy controls [mean (standard deviation) age = 24.97 (4.72) years, 17 females (57%)] underwent functional MRI scans while performing the two-step Markov decision task, which was designed to dissociate goal-directed behaviour from habitual behaviour. We employed a neurocomputational model to account for an uncertainty-based arbitration process, in which a prefrontal arbitrator (i.e. inferior frontal gyrus) allocates behavioural control to a more reliable strategy by selectively gating the putamen. We analysed group differences in the neural estimates of uncertainty of each strategy. We also compared the psychophysiological interaction effects of system preference (goal-directed versus habitual) on fronto-striatal coupling between groups. We examined the correlation between compulsivity score and the neural activity and connectivity involved in the arbitration process.

The computational model captured the subjects’ preferences between the strategies. Compared with healthy controls, patients had a stronger preference for the habitual system (*t* = −2.88, *P* = 0.006), which was attributed to a more uncertain goal-directed system (*t* = 2.72, *P* = 0.009). Before the allocation of controls, patients exhibited hypoactivity in the inferior frontal gyrus compared with healthy controls when this region tracked the inverse of uncertainty (i.e. reliability) of goal-directed behaviour (*P* = 0.001, family-wise error rate corrected). When reorienting behaviours to reach specific goals, patients exhibited weaker right ipsilateral ventrolateral prefronto-putamen coupling than healthy controls (*P* = 0.001, family-wise error rate corrected). This hypoconnectivity was correlated with more severe compulsivity (*r* = −0.57, *P* = 0.002).

Our findings suggest that the attenuated top-down control of the putamen by the prefrontal arbitrator underlies compulsivity in obsessive-compulsive disorder. Enhancing fronto-striatal connectivity may be a potential neurotherapeutic approach for compulsivity and adaptive decision-making.

## Teaser

Kim *et al.* propose that compulsivity in obsessive-compulsive disorder results from reduced top-down control of a habit controller (the putamen) by a prefrontal arbitrator. Enhancing fronto-striatal connectivity could be a promising strategy for reducing compulsivity and improving adaptive decision-making.

**See Robbins *et al*. (https://doi.org/10.1093/brain/awae133) for a scientific commentary on this article.**

## Introduction

At least one-third of patients with obsessive-compulsive disorder (OCD) are unsatisfactorily responsive to first-line therapies due to biological heterogeneity.^1,2^ As brain mechanisms could construct biotypes with homogeneous underlying pathophysiologies,^3^ neurotherapeutics guided by neurocircuit models are expected to be an alternative for non-responders.^4^ However, neurobiological evidence may not provide a useful framework for developing novel therapies unless based on a theory of the illness. While some theory-free evidence may describe its correlations with clinical symptoms, identifying intervention targets is challenging due to the existence of multiple neurobiological causes.^5^ Therefore, building theory-driven neurocircuit models is needed to develop a neurotherapeutic intervention for OCD.

Contrary to a traditional theory that states that compulsions arise as a consequence of obsessions and metacognitive deficits (e.g. overestimated credibility of one’s thoughts),^6,7^ modern neuropsychological findings propose a maladaptive habit hypothesis that considers failures in habitual controls as building blocks of compulsions.^8,9^ The latter has been supported by the observation that compulsive behaviours could arise from a long history of habits under punishment.^10,11^ Indeed, patients with OCD have difficulty ceasing repetitively trained behaviours despite devaluation of outcomes associated with the actions^12,13^; however, the failure to avoid devalued choices could also indicate disrupted goal-directed controls. The observation that goal-directed systems, which compete with habitual systems for optimal decision-making,^14^ are also disrupted in OCD has led to the theory that an imbalance between the two systems may be the basis of compulsions.^15,16^

The imbalance between the two systems can be assessed using a two-step decision task paradigm. In this paradigm, alterations in the state-transition probability elicit uncertainty about the task structure, hindering planning and enabling the dissociation of the two systems.^17^ This paradigm has been modified to manipulate additional factors that differentiate the two systems. In particular, a two-step Markov decision task incorporates a goal specificity condition, motivating subjects to represent future states.^18^ Given the condition of inducing active goal seeking, the modified task is useful in assessing dynamic adaptation processes in decision-making.^19^ Two-step decision paradigms have revealed that compulsive disorders, such as OCD, binge eating disorder and drug addiction, are less goal-oriented but more habitual (i.e. a bias towards habitual behaviours).^20,21^ In particular, impaired goal-directed planning has been shown to be associated with compulsivity.^22^ However, little is understood about how the imbalance occurs during the arbitration process between the two strategies in OCD.

A computational model of arbitration suggests that a less uncertain strategy dominates the control over behaviours of the two systems.^23^ Adopting the idea of uncertainty-based competition, Lee *et al*.^19^ proposed that the uncertainty in predictions of each strategy can be approximated by tracking the performance reliability of these systems through the history of predictions. Based on this proposal, computational neuroscience studies have identified the inferior frontal gyrus (IFG) as a prefrontal arbitrator that encodes the reliability (inverse of uncertainty) of the strategies and allocates behavioural control to a more reliable one.^19,24,25^ When the goal-directed system is deemed to be dominant over the other, the prefrontal arbitrator suppresses the posterior putamen, which is a habitual system controller involved in the estimation of the prediction error or action value of the system.^19,26^ Because OCD is characterized by enhanced intolerance of uncertainty regarding changes in goal-directed contingency,^27,28^ this uncertainty-based arbitration model would fit to test the imbalance theory in OCD. Given the crucial role of uncertainty differences in the arbitration process, the conditions of the Markov decision task provide a suitable environment for inducing transitions between the strategies by manipulating their prediction error or uncertainty.

Model-based (MB) reinforcement learning describes goal-directed behaviour in that goal-directed planning requires an internal model of task structure. In contrast, model-free (MF) learning, a trial-and-error strategy without building an explicit model of the environment, is assumed to capture habitual behaviour.^23,29^ However, goal-directed behaviour is a more complex process that involves additional tasks: retrieving contextually-relevant information from episodic memory, setting goals to appropriately infer states and actions and utilizing intrinsic motivation.^18,30^ Although current MB algorithms may not fully encompass all aspects, they are effective at assessing the competence and uncertainty in model-based predictions.

Despite earlier attempts to examine the neurochemical or morphological contributions to the imbalance between the two strategies,^20,31^ much remains unknown about how it occurs during the learning process. Using the neurocomputational model,^19^ the current study aimed to clarify the functional impairment in the dynamic arbitration mechanism behind habit bias in OCD, which may be a potential target for theory-driven non-invasive neuromodulation for compulsivity. Fronto-striatal circuitry mediates executive functions.^32^ Specifically, the ventrolateral prefrontal cortex (vlPFC) and putamen are recruited for the inhibition of repetitive behaviours, while the dorsolateral prefrontal cortex (dlPFC) and caudate are activated to execute goal-directed planning.^14,33,34^ In OCD, impairments in response inhibition or planning involve hypoactivation in the vlPFC or dlPFC area, respectively.^35-38^ While a disrupted caudate is involved in both cognitive deficits,^35,39^ the observation of hyperactivation in the putamen before avoidance of symptom-provoking stimuli indicates that this region is implicated in maladaptive habits.^40^ However, the interregional couplings within the circuitry in relation to the impairments are complex in OCD despite consistent lateral prefronto-striatal hypoconnectivity.^41,42^ Difficulties avoiding habits or executing goal-directed planning are related to vlPFC-caudate or dlPFC-putamen hypoconnectivity,^36,39,43^ while putamen connectivity with the vlPFC is disrupted in the resting-state.^41^ According to the arbitration model,^19^ there is an additional neural system that determines which of the two systems is engaged; hence, the arbitration neural system (i.e. gating the putamen by the prefrontal arbitrator as a result of uncertainty competition) is distinct from the goal-directed or habitual neural system itself. Given that the dynamic arbitration mechanism is our research interest, we focused on the IFG encoding uncertainty of strategies and the negative coupling between the prefrontal arbitrator and the putamen, which determines when the goal-directed or habitual system is engaged.

**Figure 1:**
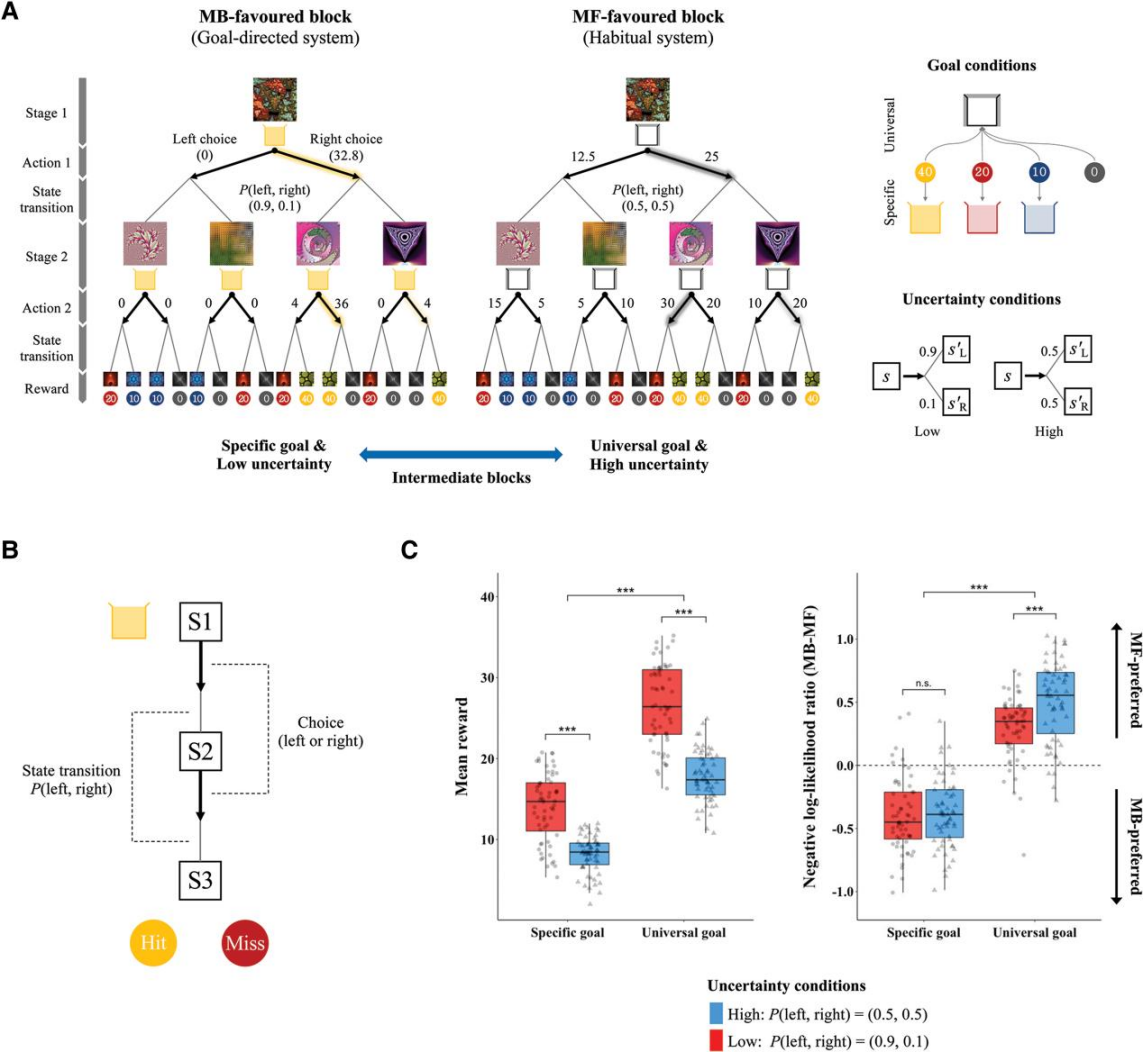
**Task paradigm dissociating goal-directed behaviour from habitual behaviour.** (**A**) In the two-step decision task, participants navigated through two sequential stages by pressing the left or right button to reach a reward stage. At this stage, they collected a coin with points to a collection box. The goal varied based on the task conditions: either matching the colour of the box (specific goal) or regardless of the box colour (universal goal). Fractal images served as visual representations of the state space. Participants made a choice (bold arrow) at the first or second stage, followed by a transition to the left or right state (via solid line) in the subsequent stage. The probability of these transitions was either *P*(left, right) = (0.9, 0.1) or *P*(left, right) = (0.5, 0.5) under low or high uncertainty, respectively. The vertical bar (*left*) outlines the trial procedure. Expected returns following each choice are denoted next to the arrows, with highlighted bold arrows indicating optimal choices expected to yield the highest returns at each state (s, s′). The task induced a preference for either goal-directed (MB) or habitual (MF) learning by manipulating the goal of behaviour and state-transition uncertainty. L = left; R = right. (**B**) A diagrammatical representation of the task procedure from Stage 1 (S1) to Stage 2 (S2) and the Reward stage (S3). Participants matched the colour of the coin with a box to obtain a reward if the box was coloured yellow, red or blue (specific goal). (**C**) *Left*: Participants could achieve greater rewards when the goal was not specifically confined (universal goal) or the transition uncertainty was low (red boxes and circle data-points) than when a specific goal or high uncertainty (blue boxes and triangle data-points) was set. *Right*: The negative log-likelihood of each model indicates how precisely the model accounts for the subjects’ behaviours (e.g. the lower the value, the more precise the prediction). The ratio (MB minus MF) of the values showed that participants preferred to use the goal-directed (MB) over the habitual (MF) learning strategy under the specific goal or low uncertainty condition and vice versa under the universal goal or high uncertainty condition.

Based on previous findings,^27,28,37,38,41^ we hypothesized that habit bias in OCD is attributed to enhanced uncertainty in the MB strategy and hypoactivation in the IFG, tracking the inverse of the uncertainty of that strategy. During the arbitration process, patients would exhibit weaker coupling between the IFG and the putamen than healthy controls (HCs) when reorienting behaviours towards the MB strategy. We also hypothesized that aberrations in the vlPFC-putamen circuit are associated with compulsivity. To test these hypotheses, we carried out task-based functional MRI (fMRI) in patients with OCD and HCs during a sequential two-step Markov decision task (Fig. 1A).^19^ To account for the arbitration process between the two strategies, we employed a computational model previously validated using data from healthy volunteers.^19^

## Materials and methods

### Participants

We recruited 35 patients with OCD and 31 HCs at the Seoul National University Hospital. The diagnosis of OCD was made by licensed psychiatrists using the Structured Clinical Interview for DSM-IV Axis-I disorders (SCID-I), patient edition.^44^ We assessed the severity of obsessive-compulsive symptoms and accompanying depression and anxiety using the Yale-Brown Obsessive Compulsive Scale (Y-BOCS) and the Hamilton Rating Scales for Depression and Anxiety (HAM-D/A).^45-47^ Of the original dataset of 66 subjects, 30 patients and 30 HCs remained after screening (Table 1 and Supplementary material, ‘Methods’ section). All participants provided written informed consent. The study was approved by the Institutional Review Board of Seoul National University Hospital (No. H-1908-208-1066) and was performed in accordance with the ethical guidelines of the Declaration of Helsinki.

### Behavioural task

We used the sequential two-step Markov decision task to assess MB and MF learning (Fig. 1A).^19^ Participants underwent two sequential decision-making stages involving pressing either the left or right button to earn points (40, 20, 10 or 0) at the reward stage. The goal was to collect as many points as possible. If they did not choose within 4 s, the task made a random choice for them and penalized their points.

In each trial, participants started at the same state. After their first and second choices, they moved to the next stage, determined by a hidden state-transition probability (Fig. 1B). They were aware that task contingencies might change during the experiment, and the state-transition probabilities were not disclosed. There was a brief interval between 1 and 4 s before each stage, and the reward stage lasted 2 s.

The task manipulated the goal of behaviour (specific or universal) and state-transition uncertainty (high or low) to differentiate the two strategies. Under the specific goal, participants aimed to match the colour (red, yellow or blue) of the coin with a coloured box to obtain a reward. The changing box colour across trials induced participants to actively consider the current state’s valuable goal (MB strategy). Under the universal goal, participants were presented with a white box and could receive a reward from any coloured coin, inducing trial-and-error learning (MF strategy).

The state-transition probabilities were set to *P*(left, right) = (0.5, 0.5) for high uncertainty and changed to *P*(left, right) = (0.9, 0.1) for low uncertainty. Relative to low uncertainty, high uncertainty made it challenging for participants to use an internal model to achieve the goal, promoting the use of the MF strategy.

Overall, the task induced a preference for the MB strategy under specific goal and low uncertainty conditions (MB-favoured block) or for the MF strategy under universal goal and high uncertainty conditions (MF-favoured block). The other two combinations of conditions induced an intermediate preference between the two strategies. Each session included the repetition of each block twice, and the sequence was randomized. Participants needed to learn about state-action-outcome associations and make optimal choices under varying conditions.

The experimental task comprised six sessions, each with around 40 trials. Prior to the experimental sessions, participants underwent a pretraining session consisting of 100 trials. This session aimed to familiarize participants with the task structure, with the state-transition probability fixed at the high uncertainty condition with the universal goal for the first 80 trials and the specific goal for the remaining trials.

### Computational model of arbitration

**Figure 2:**
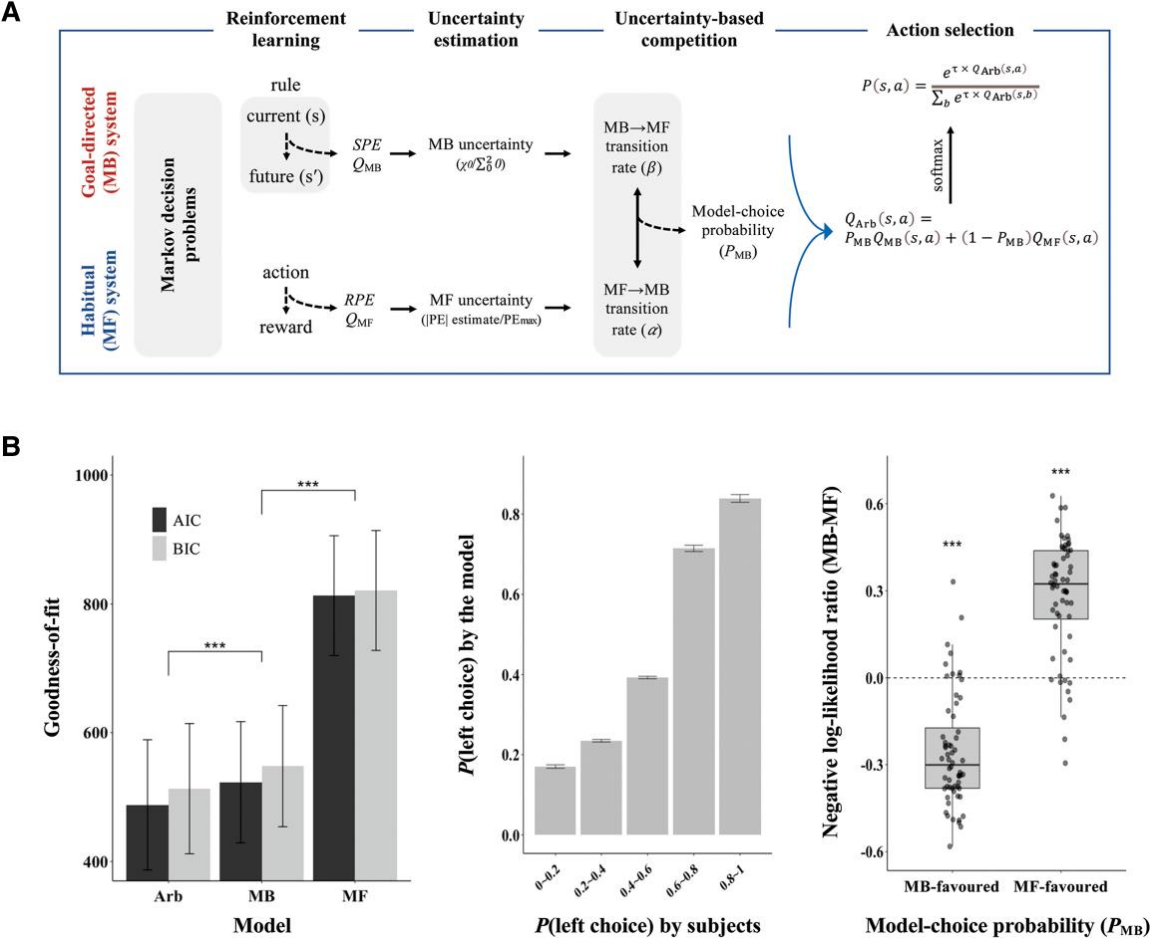
**The arbitration process between goal-directed and habitual decision-making.** (**A**) Given states (*s*) and actions (*a*), the computational model computed variables responsible for the arbitration process between the goal-directed (MB) and habitual (MF) learning strategies in the following framework. During reinforcement learning, the action values (*Q*_MB_ and *Q*_MF_) of the MB and MF learners were updated according to the state-prediction error (SPE) and reward-prediction error (RPE), respectively. The uncertainty of MB and MF learning was estimated given the prediction error history of each strategy. The arbitrator updated the probability of choosing MB over MF (*P*_MB_) by comparing the uncertainty between the two strategies. The model selected an action *a* with the probability *P*(*s, a*) computed using the value integrated from both systems (*Q*_Arb_). (**B**) Model validation. *Left*: The arbitration model (Arb) outperformed the models without arbitration (MB or MF alone) in explaining subjects’ adaptive decision-making. The results of the model comparisons were consistent across two metrics [Akaike information criterion (AIC) and Bayesian information criterion (BIC)] of goodness-of-fit. *Middle*: The proportion of the arbitration model’s left choice increased with the probability of the same choice by subjects in the same state, which demonstrated that the model captured variability in subjects’ choice behaviour. *Right*: The model-choice probability (*P*_MB_) estimated by the model captured the subjects’ preference between the two strategies: the negative or positive log-likelihood ratio when the model favoured the MB (*P*_MB_ > 0.5) or MF strategy (*P*_MB_ < 0.5), respectively. ***P < 0.001, *ns* = not significant.

To account for how imbalance occurs during the arbitration process in OCD, we employed a computational model of arbitration that makes an inference about the prediction uncertainty of each strategy based on its history of prediction error, allocating behavioural control to a more reliable strategy.^19^ The model computed variables responsible for the arbitration via the following framework (Fig. 2A). In Step 1, we employed MB and MF reinforcement learning, updating action values iteratively using Bellman’s equation. The MB learner used FORWARD learning and BACKWARD planning to update its action value (*Q*_MB_) based on the state-prediction error (SPE), an error in the prediction of the state-transition probability.^48^ The action value (*Q*_MF_) of the MF learner was updated through the SARSA algorithm, considering the reward-prediction error (RPE).^29^ In Step 2, the arbitration model estimated the uncertainty of each learning given the history of prediction errors. For MB learning, we used hierarchical Bayesian inference for the prediction uncertainty (*χ*_MB_) given the SPE history.^19^ For MF learning, we employed the Pearce-Hall associability learning theory to estimate the uncertainty (*χ*_MF_) based on the absolute value of the RPE.^19,49^ Additionally, the maximum uncertainty of the two strategies was calculated to indicate the uncertainty of whichever strategy was the most uncertain for each trial across the arbitration process. In Step 3, we implemented uncertainty-based competition between MB and MF learning by using a dynamic two-state transition model.^50^ Transition rates between the two systems were adjusted based on their respective uncertainty estimates. For example, the transition rate from MF to MB learning (*α*) or vice versa (*β*) was a function of *χ*_MF_ or *χ*_MB_, respectively:


(1) $$\alpha \;=\;A_{\alpha }/[1\;+\;e^{B_{\alpha }(1\;-\;\chi_{\mathrm{MF}})}]$$

(2) $$\beta \;=\;A_{\beta }/[1\;+\;e^{B_{\beta }(1\;-\;\chi_{\mathrm{MB}})}]$$

where *A_α_* (or *A_β_*) is the amplitude of the MF→MB (or MB→MF) transition rate function and *B_α_* (or *B_β_*) is the steepness of the corresponding transition rate function. The probability of choosing MB over MF strategy (*P*_MB_) was dynamically updated through between-strategy competition depending on the difference in their uncertainty (e.g. if the model determines that the MB strategy is more uncertain than the other, the MF strategy is likely to be chosen instead):


(3) $$dP_{MB}/dt\;=\;\alpha (1\;-\;P_{MB})\;-\;\beta P_{MB}$$

Last, we computed the action value of the arbitration system (*Q*_Arb_) by integrating the action values of the two strategies in a weighted average manner by the *P*_MB_. The arbitrator selected an action through the softmax (likelihood) function, taking as input the *Q*_Arb_, with an inverse temperature parameter (*τ*) controlling decision-making. We provide details of the algorithms in the Supplementary material.

### Model fitting and evaluations

We fitted the model parameters to the observed behaviours for each individual by minimizing the negative log-likelihood of the model’s selection probability for an action *a* chosen by a subject [−∑log*P*(*s*,*a*)], summed across trials.

Using the Wilcoxon signed rank test, we compared the goodness-of-fit (i.e. sum of the negative log-likelihood) of three different models explaining behaviour on the decision task: one with the arbitration process between the MB and MF systems and two without it (i.e. solely by the MB or MF learning system). For a parameter recovery analysis, we generated behavioural data using the model with the optimized model parameters and tested Pearson correlations between the original and recovered parameters. To demonstrate the predictability of the model, we compared the probability that participants chose the left action in each state and the proportion of the left choice by the model in the same states.

### Model-based functional MRI analysis

We analysed the fMRI data using Statistical Parametric Mapping version 12 (www.fil.ion.ucl.ac.uk/spm/). To test the effects of the task conditions, we constructed a first general linear model (GLM) including eight types of impulse events at the onsets of Stages 1 and 2 (decision phase) as well as Stage 3 (reward feedback phase) throughout the four blocks (GLM 1). The stick function in the decision or reward feedback phase was parametrically modulated by response speed or obtained reward amount, respectively (Table 2).

**Table 1:** Demographics and clinical characteristics of participants

Variables	OCD (*n* = 30)		HCs (*n* = 30)		OCD versus HCs		
	*n*	%	*n*	%	*χ2*	df	*P*-value
Female^a^	12	40	17	57	1.67	1	0.20
Left-handedness	2	7	4	13	0.74	1	0.39
Symptom dimension^b^							
Contamination/cleaning	11	37	−	−	−	−	−
Symmetry/ordering	1	3	−	−	−	−	−
Aggression/checking	24	80	−	−	−	−	−
Sexual/religious	1	3	−	−	−	−	−
Miscellaneous	12	40	−	−	−	−	−
Treatments at the time of study							
SSRIs	27	90	−	−	−	−	−
Anxiolytics	15	50	−	−	−	−	−
CBT^c^	0	0	−	−	−	−	−

	Mean	SD	Mean	SD	*t*	df	*P*-value
Age, years	26.93	6.23	24.97	4.72	1.38	58	0.17
Intelligence quotient	113.00	13.55	112.93	8.82	0.02	58	0.98
Education, years	14.67	1.75	14.97	1.97	−0.62	58	0.54
Y-BOCS score							
Total	22.10	5.28	−	−	−	−	−
Obsession	11.50	3.26	−	−	−	−	−
Compulsion	10.60	2.92	−	−	−	−	−
HAM-D score	7.63	6.11	−	−	−	−	−
HAM-A score	7.10	5.48	−	−	−	−	−
Duration of illness, years	7.69	4.90	−	−	−	−	−

CBT = cognitive behavioural therapy; df = degrees of freedom; HAM-A = Hamilton Anxiety Rating Scale; HAM-D = Hamilton Depression Rating Scale; HC = healthy control; OCD = obsessive-compulsive disorder; SD = standard deviation; SSRI = selective serotonin reuptake inhibitor; Y-BOCS = Yale-Brown Obsessive Compulsive Scale.

^a^The participants reported that their gender identity was the same as their sex assigned at birth.

^b^Measured with the Y-BOCS symptom checklist.

^c^CBT experience for at least 1 year.

**Table 2:** Designs of the general linear models for functional MRI analyses

	Condition	Onset	Parametric modulator
GLM 1	1	Stage 1, Stage 2 (Block 1)	Response speed
	2	Stage 1, Stage 2 (Block 2)	Response speed
	3	Stage 1, Stage 2 (Block 3)	Response speed
	4	Stage 1, Stage 2 (Block 4)	Response speed
	5	Reward stage (Block 1)	Obtained reward
	6	Reward stage (Block 2)	Obtained reward
	7	Reward stage (Block 3)	Obtained reward
	8	Reward stage (Block 4)	Obtained reward
GLM 2	1	Stage 1, Stage 2, Reward stage	State-prediction error, reward-prediction error
GLM 3	1	Stage 1, Stage 2, Reward stage	*χ*_MB_, *χ*_MF_ (or max(*χ*_MB_, *χ*_MF_) for replication test)
GLM 4	1	Action 1, Action 2	*Q*_MB_ (chosen), *Q*_MB_ (chosen), chosen minus unchosen *Q*_Arb_

Action 1 = timing of the first choice made; Action 2 = timing of the second choice made; Block 1 = block with specific goal and low uncertainty conditions; Block 2 = block with specific goal and high uncertainty conditions; Block 3 = block with universal goal and high uncertainty conditions; Block 4 = block with universal goal and low uncertainty conditions; GLM = general linear model; HC = healthy control; k_E_ = cluster extent; MB = model-based learning (goal-directed behaviour); MF = model-free learning (habitual behaviour); OCD = obsessive-compulsive disorder; *Q*_Arb_ = action value of the arbitration system; *Q*_MB_ = action value of MB learning; *Q*_MF_ = action value of MF learning; Reward stage = timing of a reward feedback presented; Stage 1 = timing of the first stage presented (making a first decision); Stage 2 = timing of the second stage presented (making a second decision); *χ*_MB_ = uncertainty of MB learning; *χ*_MF_ = uncertainty of MF learning.

To investigate the neurocomputational mechanism during the arbitration process, we also designed three other subject-level GLMs, each of which included the following computational variables as parametric modulators: SPE and RPE (GLM 2), uncertainty estimates of the MB and MF strategies (GLM 3), and the *Q*_MB_ and *Q*_MF_ for chosen actions and the *Q*_Arb_ difference between chosen and unchosen actions (GLM 4). In GLMs 2 and 3, impulse events at the onsets of all three stages (Stage 1, Stage 2 and the Reward stage) were modulated by the computational variables of prediction errors or uncertainty estimates, while GLM 4 modulated impulse events when the first and second choices were made (Actions 1 and 2) by the action values (Table 2). All the GLMs included the motion parameters as covariates. Given that the IFG selectively gates the putamen to guide goal-directed or habitual actions,^19,26^ we analysed the psychophysiological interaction (PPI) effects of system preference (i.e. *P*_MB_) on fronto-striatal coupling. For the PPI analyses, we extracted physiological signals from 5 mm radius spherical regions of interest: the bilateral IFG seeds (Supplementary Table 2) found to encode the maximum uncertainty of both strategies and the bilateral putamen seeds [Montreal Neurological Institute (MNI) coordinates ±27, −13, 4] involved in MF learning.^51^ For group comparisons, we used the putamen seeds to identify a cortical target for viable application of noninvasive neuromodulation. The PPI GLMs included the interaction term as a covariate of interest while controlling for the physiological and psychological terms.

### Statistical analyses

For the group comparisons of behaviours, we conducted three-way mixed-design ANOVAs from the SciPy statistics module (www.scipy.org/) with the goal and uncertainty conditions as within-subject factors for the mean reward and the *P*_MB_, which was followed by *post hoc* two-sample *t*-tests. For the two-sample *t*-tests of behaviours and the model parameters, we applied Bonferroni correction to obtain family-wise error rate corrected *P* (*P*_FWER_) < 0.05 in the comparisons of the behavioural variables (*P* < 0.012), model parameters (*P* < 0.008) or computational variables (*P* < 0.012) between groups. For subsequent behavioural correlation analyses, we chose the MB-favoured or MF-favoured block to robustly compare behavioural variables underlying decision-making either during goal-directed or habitual learning.

In group-level fMRI analyses, we defined a significant difference if a cluster survived the cluster-extent threshold of *P*_FWER_ (cluster *P*_FWER_) < 0.05 among clusters formed by suprathreshold voxels (uncorrected *P* < 0.001). We labeled the brain regions using the Automated Anatomical Labeling 3 and NeuroSynth atlases.^52,53^ To address concerns about the moderate sample size, we conducted *post hoc* non-parametric permutation tests (cluster-forming *t* < 0.001, 10 000 repetitions) of the neurocomputational differences between groups using SnPM 13 (warwick.ac.uk/snpm/).

We performed Spearman’s correlation analyses between the mean reward/model parameters and *P*_MB_ and between the behavioural/clinical outcomes and the neural correlates/connectivities. To address concerns about the influences of clinical heterogeneity, we controlled for comorbidities and drug doses (selective serotonin reuptake inhibitors and anxiolytics) in the correlation analyses with clinical variables.

## Results

### Effects of task conditions dissociating the MB from the MF strategy

The effects of task conditions were evaluated using two-way repeated measures ANOVA. As presented in Fig. 1C, we observed effects of an interaction between the goal type and uncertainty level on the mean reward (*F* = 35.27, *P* < 0.001) and the negative log-likelihood ratio (MB minus MF) (*F* = 20.29, *P* < 0.001). The latter measure was used to indicate which strategy subjects were more likely to adopt, as a lower negative log-likelihood of a strategy means a higher likelihood that their behaviour could be explained by that strategy. There were significant simple main effects of all task conditions on the behavioural outcomes (*P*s < 0.001) except for the effect of uncertainty on the negative log-likelihood ratio during the specific goal condition (*P* = 0.559).

### Computational model accounting for adaptive decision-making

Goodness-of-fit metrics estimated using the sum of the negative log-likelihood across trials showed that our computational model of uncertainty-based arbitration outperformed the models without arbitration in explaining subjects’ adaptive decision-making (*P*s < 0.001; Fig. 2B and Supplementary Table 1), as previously demonstrated.^19,24^ The model effectively accounted for the participants’ behaviours in the decision task (Fig. 2B), and its parameters were successfully recovered from the behaviour (*P*s < 0.001; Supplementary material, ‘Results’ section). As expected, the arbitration model captured the participants’ preference between the MB and MF strategies (Fig. 2B). One-sample *t*-tests of the negative log-likelihood ratio (MB minus MF) averaged across trials when the arbitration model favoured the MB (*P*_MB_ > 0.5) or the MF strategy (*P*_MB_ < 0.5) showed a more negative value when the model favoured the MB strategy (*t* = −10.32, *P* < 0.001) and vice versa when the other strategy was preferred (*t* = 10.60, *P* < 0.001).

### Biased preference of the MF over the MB strategy and suboptimal decision-making in OCD

**Figure 3:**
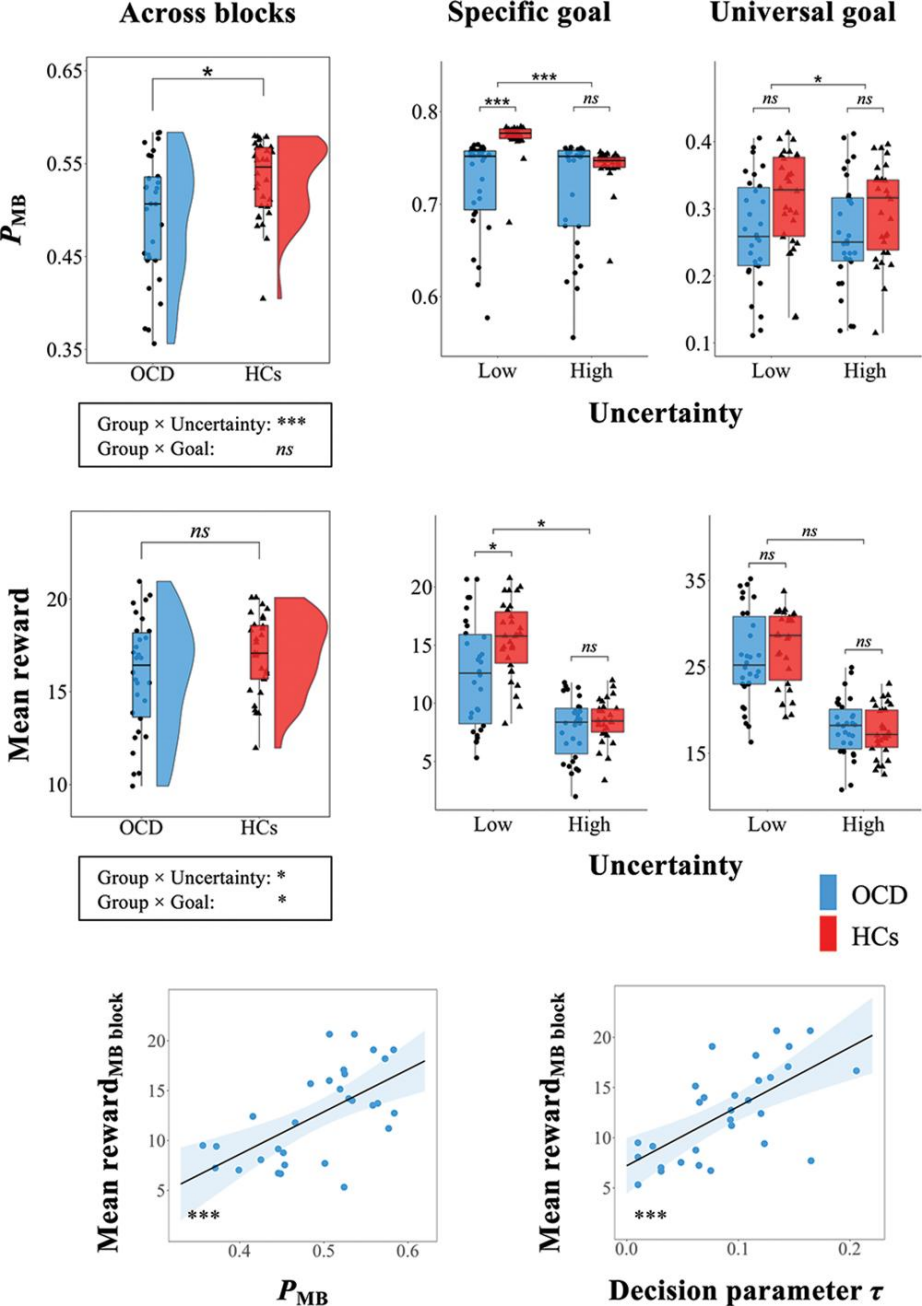
**Habit bias and its association with inefficient goal-directed behaviours in obsessive-compulsive disorder.** *Top*: As a result of the uncertainty-based competition between the two strategies, patients with obsessive-compulsive disorder (OCD) exhibited a shift away from the goal-directed (MB) towards the habitual (MF) learning strategy (lower *P*_MB_) than healthy controls (HCs), especially during the MB-favoured block. *Middle*: Patients obtained smaller mean rewards when goal-directed behaviours were encouraged (MB-favoured block) than HCs, while the behavioural performance was comparable between groups when habitual actions were induced (MF-favoured block). *Bottom*: Impaired goal-directed behaviour in patients was associated with preference bias towards the MF strategy and a decreased reliance on the value of choices in decision-making. *P_FWER_ < 0.05, ***P_FWER_ < 0.001, *ns* = not significant. FWER = family-wise error rate corrected.

As presented in Fig. 3, patients were more likely to prefer the MF over MB strategy (i.e. lower *P*_MB_) across blocks than HCs (*t* = −2.88, *P* = 0.006). For *P*_MB_, the mixed-design ANOVA showed a significant interaction effect between group and uncertainty condition (*F* = 44.03, *P* < 0.001). This interaction remained significant within both the specific (*F* = 63.93, *P* < 0.001) and universal goal conditions (*F* = 6.98, *P* = 0.011). We observed a simple main effect of group during the MB-favoured block (specific goal and low uncertainty), which indicated lower *P*_MB_ in patients than in HCs (*t* = −5.10, *P* < 0.001). The mean rewards across blocks were comparable between groups (*t* = −1.60, *P* = 0.116). The mixed-design ANOVA of mean rewards showed significant effects of Group × Uncertainty interaction (*F* = 4.42, *P* = 0.040) and Group × Goal interaction (*F* = 4.15, *P* = 0.046). The Group × Uncertainty interaction remained significant within the specific goal condition (*F* = 6.76, *P* = 0.012). We observed a simple main effect of group during the MB-favoured block, indicating smaller mean rewards in patients than in HCs (*t* = −2.91, *P* = 0.005). On the other hand, the PMB (*t* = −1.65, *P* = 0.105) and mean reward (*t* = 0.52, *P* = 0.603) were comparable between groups during the MF-favoured block.

The computational model elucidated how this preference bias resulted from the uncertainty-based competition process between the two strategies. Among the computational variables, the estimated uncertainty in the MB strategy was higher in patients than in HCs (*t* = 2.72, *P* = 0.009), while that in the MF strategy was comparable between groups (*t* = 0.47, *P* = 0.644). Owing to the higher uncertainty in the MB over the MF strategy, patients had a higher rate of MB→MF transition (*β*) than HCs did (*t* = 3.19, *P* = 0.003) at the uncertainty-based competition step, while that of the opposite transition (*α*) was comparable between groups (*t* = −0.39, *P* = 0.701). Based on this imbalance, the arbitration system had difficulty making value-based decisions (i.e. lower decision parameter *τ*) in patients than in HCs (*t* = −3.01, *P* = 0.004; Supplementary material, ‘Results’ section). Within OCD patients, a smaller amount of reward was positively correlated with a lower *τ* (*ρ* = 0.65, *P* < 0.001) and *P*_MB_ (*ρ* = 0.60, *P* < 0.001) (Fig. 3).

### Activity of fronto-striatal regions during goal-directed or habitual behaviour in OCD

**Figure 4:**
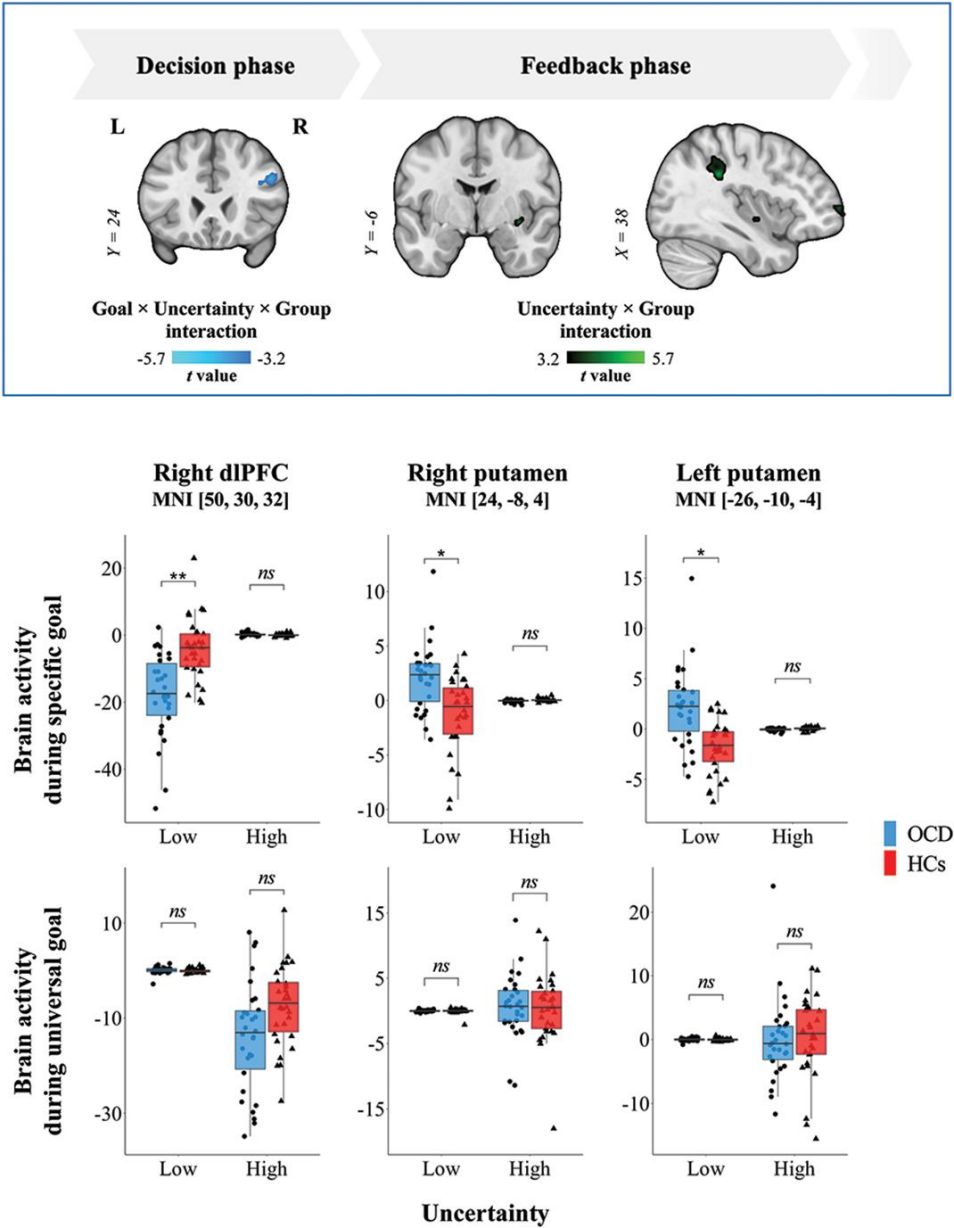
**Activity of fronto-striatal regions during goal-directed or habitual behaviour in obsessive-compulsive disorder.** In the two-step decision task, each trial comprised two phases: decision (Stages 1 and 2) and reward feedback (Stage 3). In obsessive-compulsive disorder (OCD), there were aberrant responses of the dorsolateral prefrontal cortex (dlPFC) to both goal and uncertainty conditions in the decision phase, while those of the putamen and inferior parietal lobe to uncertainty condition were observed in the feedback phase (*top*). In particular, patients exhibited hypoactivity in the dlPFC and increased recruitment of the putamen within the goal-directed (MB)-favoured block compared with healthy controls (HCs) (*bottom*). *P_FWER_ < 0.05, **P_FWER_ < 0.01, *ns=* not significant. FWER = family-wise error rate corrected; MNI = Montreal Neurological Institute.

To ascertain the presence of brain dysfunctions during goal-directed or habitual actions in the regions previously reported in OCD, we analysed mixed-design ANOVAs with the goal and uncertainty conditions as within-subject factors, which was followed by *post hoc* comparisons of neural activity during each block between groups. There was a significant Goal × Uncertainty × Group interaction effect in the right supramarginal gyrus and dlPFC (cluster *P*s_FWER_ ≤ 0.001) in the decision phase (Fig. 4). We found hypoactivity in the right dlPFC (MNI 50, 30, 32; *t* = −3.9) within the MB-favoured block in patients compared with HCs. In the reward feedback phase, there was a significant Uncertainty × Group interaction effect in the right putamen and inferior parietal lobe (cluster *P*s_FWER_ < 0.05). Patients exhibited hyperactivity of the right putamen (MNI 24, −8, 4; *t* = 3.9) and left putamen (MNI −26, −10, −4; *t* = 5.3) within the MB-favoured block compared with HCs.

### Aberrant brain functions and connectivities during the arbitration between the strategies in OCD

**Figure 5:**
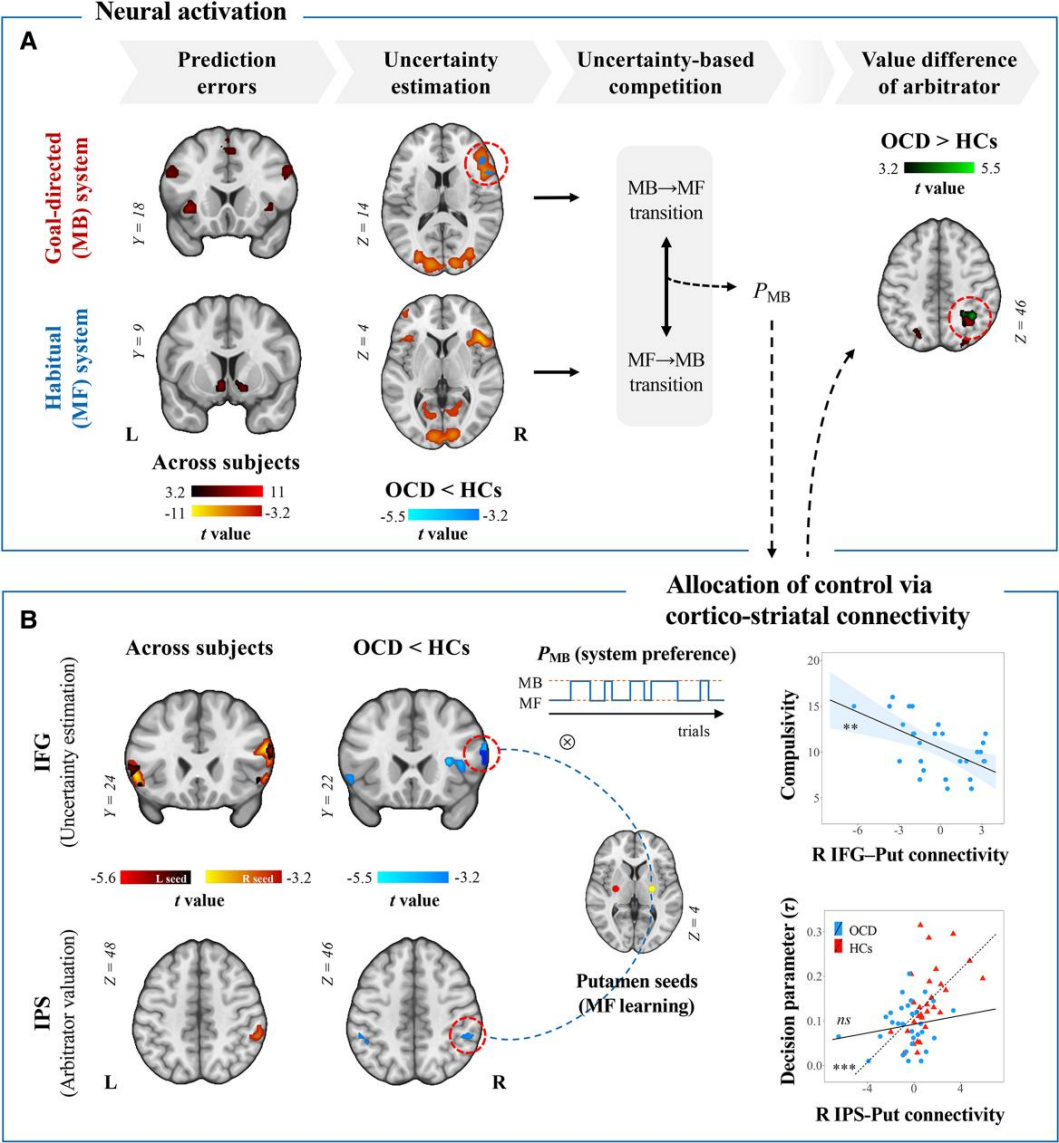
**Ventrolateral prefronto-putamen circuit involved in the arbitration process and compulsivity in obsessive-compulsive disorder.** (**A**) During the arbitration process between the goal-directed (MB) and habitual (MF) learning strategies, patients did not exhibit dysfunctions in regions encoding prediction errors or action values of the MB and MF strategies. Instead, they showed hypoactivity in the right inferior frontal gyrus (IFG) encoding uncertainty of the MB strategy and hyperactivity in the intraparietal sulcus (IPS) accumulating evidence in favour of a particular decision compared with healthy controls (HCs). (**B**) When the MB strategy was preferred over the MF strategy, the putamen involved in MF learning was negatively coupled with the IFG encoding the uncertainty of the strategies. Given that uncertainty-based competition resulted in a preference bias towards the MF strategy (i.e. lower *P*_MB_), patients had weaker right ipsilateral IFG-putamen connectivity than HCs. Weaker fronto-striatal connectivity was correlated with higher Yale-Brown Obsessive Compulsive Scale (Y-BOCS) compulsivity scores in patients with obsessive-compulsive disorder (OCD). Additionally, the IPS-putamen negative coupling engaged in redirecting behaviours towards the MB strategy was attenuated in patients compared with HCs. Parieto-striatal connectivity was no longer associated with the extent to which decision-making relied on the value of choices in patients. **P_FWER_ < 0.01, ***P_FWER_ < 0.001, *ns =* not significant. L = left; R = right.

To investigate brain impairments contributing to the imbalance between the two systems, we performed model-based fMRI analysis using the computational variables responsible for the arbitration as parametric modulators in GLMs. We first replicated previous findings of neural correlates of the computational variables using one-sample *t*-tests (cluster *P*s_FWER_ < 0.012).^19,24^ As presented in Supplementary Table 2 and Fig. 5A, we observed neural signals encoding the SPE (insula and dorsolateral prefrontal cortex), RPE (nucleus accumbens and putamen), *Q*_MB_ (medial prefrontal cortex and premotor cortex) and *Q*_MF_ (supplementary motor area and putamen) for chosen actions. The bilateral IFG signals (MNI −50, 20, 6 and MNI 50, 18, 8) encoded the maximum uncertainty of MB and MF learning as well as that of each strategy. The *Q*_Arb_ difference between chosen and unchosen actions was represented by the intraparietal sulcus (IPS) and cerebellar crus II. From the PPI analyses using the IFG and putamen seeds, we replicated that when the MB strategy was deemed to be dominant, the right IFG signal was negatively coupled with the right putamen signal involved in MF learning (Fig. 5B). Extended from the fronto-striatal network, the right putamen was also negatively coupled with the IPS, temporoparietal junction and cerebellar posterior lobe, while the right IFG was positively coupled with the premotor cortex.

We then compared the parameter estimates from the GLMs between groups using independent two-sample *t*-tests. While there were comparable neural activities encoding prediction errors or state-action values of each strategy between groups (Table 3), patients with OCD exhibited hypoactivity in the right IFG encoding the uncertainty of the MB strategy and hyperactivity in the right IPS encoding the *Q*_Arb_ difference compared with HCs (cluster *P*s_FWER_ ≤ 0.001; Fig. 5A). When reorienting towards the MB strategy, patients exhibited weaker negative couplings than HCs between the right putamen and bilateral IFG (cluster *P*s_FWER_ ≤ 0.001), which overlapped with the region encoding the maximum uncertainty (Fig. 5B). Additionally, putamen connectivities with the bilateral IPS (cluster *P*s_FWER_ < 0.005), which were found to encode the value difference of the arbitration system, were weaker in patients than in HCs. Functional brain differences were consistently observed from the nonparametric permutation tests (Supplementary Table 3).

**Table 3:** Aberrant brain mechanisms during the arbitration process between goal-directed and habitual systems in obsessive-compulsive disorder

Brain region	MNI coordinates, mm	Cluster size (*k_E_*)	OCD versus healthy controls		
			*t* ^a^	df	Cluster *P*_FWER_
**Main effect General Linear Model 2: prediction error estimation**					
State-prediction error					
ns	−	−	−	−	−
Reward-prediction error					
ns	−	−	−	−	−
**Main effect General Linear Model 3: uncertainty estimation**					
Uncertainty of MB learning (*χ*_MB_)					
Right IFG	56, 16, 14 (BA 44)	341	−3.7	58	0.001
Uncertainty of MF learning (*χ*_MF_)					
ns	−	−	−	−	−
**Main effect General Linear Model 4: value estimation**					
Chosen action value of MB learning (*Q*_MB_)					
ns	−	−	−	−	−
Chosen action value of MF learning (*Q*_MF_)					
ns	−	−	−	−	−
Value difference of arbitration system (chosen minus unchosen *Q*_Arb_)					
Right IPS	36, −50, 46 (BA 40)	342	5.5	58	<0.001
**Psychophysiological interaction in general linear models**					
Connectivity of left putamen^b^ when choosing MB over MF strategy (*×P*_MB_)					
ns	−	−	−	−	−
Connectivity of right putamen^b^ when choosing MB over MF strategy (*×P*_MB_)					
Left IFG	−46, 8, 20 (BA 44)	279	−5.3	58	<0.001
Right IFG	60, 22, 20 (BA 45)	143	−4.4	58	0.001
Left cerebellar crus II	−24, −78, −50 (BA −)	223	−4.0	58	<0.001
Left IPS	−48, −52, 58 (BA 40)	120	−4.0	58	0.004
Right IPS	50, −42, 40 (BA 40)	178	−3.9	58	<0.001

BA = Brodmann area; df = degrees of freedom; FWER = family-wise error rate corrected; HC = healthy control; IFG = inferior frontal gyrus; IPS = intraparietal sulcus; k_E_ = cluster extent; MB = model-based learning (goal-directed behaviour); MF = model-free learning (habitual behaviour); MNI = Montreal Neurological Institute; OCD = obsessive-compulsive disorder; *P*_MB_ = probability of choosing the MB over MF strategy.

^a^Positive/negative *t*-value indicates stronger/weaker activation or connectivity in patients than in healthy controls.

^b^First eigenvariate of the left/right putamen signal [from a 5 mm radius sphere at MNI ±27, −13, 4] implicated in MF learning.^49^

The right ipsilateral IFG-putamen hypoconnectivity was negatively correlated with the Y-BOCS compulsion score in OCD patients (*r* = −0.57, *P* = 0.002; Fig. 5B). Despite no association with any clinical outcome, parieto-striatal decoupling was related to disrupted value-based decision-making (Fig. 5B). The correlation between right ipsilateral IPS-putamen connectivity and the decision parameter *τ* was significant within HCs (*ρ* = 0.65, *P* < 0.001) but not within patients (*ρ* = 0.06, *P* = 0.736).

## Discussion

The present study aimed to identify whether a disruption in the arbitration process via the vlPFC-putamen circuit underlies the imbalance (less goal-oriented but more habitual behaviours) in OCD. Using the computational model of arbitration, we showed that habit bias in OCD was attributed to impaired goal-directed learning, which was more uncertain than habitual learning. We found that the brain mechanisms behind the imbalance between the strategies were impairments in the arbitration circuit, which allocated behavioural control depending on the difference in uncertainty between them. Patients exhibited hypoactivity in the IFG tracking the inverse of uncertainty of the MB but not of the other strategy. When reorienting towards the MB strategy, the IFG was negatively coupled with the putamen involved in MF learning. However, this top-down control by the prefrontal arbitrator was attenuated in OCD. Patients with weaker vlPFC-putamen connectivity suffered more severe compulsive symptoms. These findings support the hypotheses of the study and suggest that the vlPFC-putamen circuit is a potential target for theory-driven neuromodulation for compulsivity.

The hypothesis of an imbalance between goal-directed and habitual systems is a long-standing question in the literature on OCD. It has been primarily tested using two task paradigms: outcome devaluation and contingency degradation.^12,13,39,54^ The former is useful for observing failures of avoidance from devalued choices, while the latter is useful for testing reduced sensitivity to action-outcome contingencies. These behavioural studies successfully indicate impairments in both goal-directed and habitual systems; however, little is understood about how habitual actions become abnormally dominant during goal-directed learning in this disorder.^55^ Meanwhile, it has been suggested that an additional system exists to determine which of the two systems is engaged.^16,19^ We propose that this phenomenon could be elucidated by employing the uncertainty-based arbitration model between them in the context of the two-step decision task. This computational model makes inferences about the uncertainty of each strategy based on the history of its prediction errors and determines that the control is given to a more reliable strategy.^19^ Thus, this model is useful for testing whether a discrepancy in the prediction uncertainty between the strategies contributes to the imbalance. Consistent with previous findings,^20,22,56^ we observed that patients with OCD were more likely to shift away from goal-directed behaviour towards habitual behaviour. The current study further revealed that habit bias in patients was attributed to more uncertain predictions in goal-directed learning than in habitual learning. Such a difference in uncertainty could impede the development of goal-directed decision-making, instead contributing to the perpetuation of habitual actions in OCD.

At the neural level, the imbalance theory of OCD has been examined mainly in fronto-striatal circuitry involved in executive controls. Functional neuroimaging studies using relevant tasks have demonstrated that both goal-directed and habitual neural systems are disrupted. Reduced recruitment of dorso- and ventrolateral prefrontal cortices is related to impairments in goal-directed planning and response inhibition in OCD.^35-37^ Consistent with the literature, we observed hypoactivity in the dlPFC during the decision phase in patients when goal-directed control was demanded. The lateral prefrontal cortices mediate executive functions in collaboration with striatal regions. In OCD, hyperactivity of the putamen reflects difficulty in avoiding situations that provoke habits.^40^ This phenomenon was similarly observed in the present study: a hyperactive putamen in patients with OCD was observed when they were cued with a demand to flexibly shift away from a current decision-making strategy. In line with previous findings that the putamen is decoupled from the lateral prefrontal cortices in OCD,^36,41^ our findings suggest a pivotal role of the putamen in both goal-directed and habitual controls. While a previous study using the outcome devaluation paradigm determined a key role of the caudate in failures to avoid habits in OCD,^39^ we did not observe aberrations in this region. We posit that this inconsistency may arise because the two-step task paradigm does not overtrain habitual responses that hinder the translation of explicit contingencies into goal-directed actions and the associated function of the caudate.

Since it has been suggested that there is an additional neural system determining which of the two systems is engaged,^19,57^ researchers have hypothesized that functional impairments in the neurocircuit involved in arbitration may provide evidence to reconcile the literature suggesting disruptions in goal-directed or habitual neural systems in OCD.^8,16,31^ To test this hypothesis, we used the uncertainty-based arbitration model, which is useful for exploring the neural systems responsible for the two strategies and the balance of control between the respective systems. Using model-based fMRI analysis, the present study replicated the finding that IFG encoding of uncertainty in learning strategies selectively gates the putamen involvement in habitual learning to efficiently guide goal-directed or habitual actions.^19,26^ This fronto-striatal negative coupling when reorienting behaviours from the MF towards the MB strategy is suggested as the neural system of arbitration between the two strategies. In OCD, this IFG-putamen coupling was aberrantly attenuated when patients had difficulty shifting away from habitual towards goal-directed decision-making. Conversely, the positive coupling between the IFG and premotor cortex, responsible for encoding the action value of the MB strategy, remained intact in patients. Furthermore, this fronto-striatal hypoconnectivity was associated with more severe compulsivity. Previous studies have provided evidence of volumetric and neurochemical associations with a bias towards habitual learning in compulsive disorders by using the two-step task.^20,31^ Our neurocomputational evidence adds explicit evidence to explain how imbalance occurs during the learning process. A recent finding of less effective resting-state vlPFC-to-putamen connectivity in OCD patients indicated that disrupted top-down control by the prefrontal arbitrator may remain in the task-free condition.^58^ Exploiting task-based fMRI, the present study identified sample-specific maps of the brain dysfunctions, thereby enhancing the precision of a model for neurotherapeutic intervention. Our neurocomputational findings extend the previous understanding of the association between an impaired arbitration mechanism and compulsivity from the behavioural to the brain level.^56^ Therefore, we suggest that the uncertainty-based arbitration mechanism via the vlPFC-putamen circuit underlies the imbalance between the two strategies in OCD and serves as a biomarker for compulsions.

Before the allocation of controls, patients with OCD exhibited hypoactivity in the IFG when this region tracked the inverse of uncertainty (i.e. reliability) of MB learning. This brain alteration in the present study was in line with the behavioural findings of enhanced uncertainty about the strategy. According to the uncertainty-based arbitration model,^19,57^ the brain allocates behavioural control to a more reliable strategy. Therefore, our findings suggest that the prefrontal arbitrator exerted less regulation over the putamen in patients when the MB strategy was deemed to be dominant, possibly because their recognition of this strategy was less reliable. Hypoactivity in the vlPFC has been consistently observed in association with deficient adaptive control in OCD. In addition to its role in impairments related to inhibiting context-irrelevant responses and attentional set shifting,^37,42,59^ the present study highlights the importance of vlPFC function estimating the reliability of decision-making strategies in the pathophysiology of OCD.

The brain computes the value of each decision option and integrates them to decide for value-based decision-making. The IPS accumulates evidence in favour of a particular decision from the difference between two competing factors (e.g. costs and benefits) until a decision boundary is reached.^60^ Similarly, in this study, the IPS integrated the neural signals of action values of MB (medial prefrontal cortex) and MF systems (putamen) into a composite value. This integration was achieved through dynamically adjusting the weights between the dichotomous systems based on the difference in uncertainty. When the MB strategy was adopted, the IPS downregulated the transmission of the putamen signal of MF learning. This IPS–putamen negative coupling may lessen the engagement of the MF system in the integrated system to redirect behaviours towards the MB system. In OCD, parieto-striatal connectivity was attenuated and was no longer associated with the extent to which decision-making relied on the value of choices. While the neural system involved in value integration in the IPS was disrupted in patients, the valuation of each goal-directed and habitual neural system remained intact. Thus, our findings suggest that the shift away from the goal-directed system in OCD can be attributed to the impaired integration process resulting from the difference in uncertainty estimation between the strategies rather than the value learning of each strategy.

The unsatisfactory response rate of first-line treatments in OCD has drawn attention to developing alternative treatments based on well-established neurocircuit models.^61^ Non-invasive neuromodulation has been applied to normalize maladaptive behaviours in this disorder.^62^ Given that the imbalance between the two systems is attributed to the difference in uncertainty, we propose the vlPFC as a viable target for mitigating the bias and compulsivity. Neuromodulation studies have demonstrated that excitation or inhibition of the vlPFC could induce a preference for goal-directed or habitual actions, respectively.^63,64^ Its potential as an effective therapeutic target could be inferred from findings that deep brain stimulation indirectly affects the vlPFC via the fronto-basal ganglia connectome to enhance cognitive control in OCD.^65,66^ Because neuromodulation outcomes can be improved by connectivity-based targeting,^67^ enhancing vlPFC-putamen connectivity is expected to be more precise at alleviating compulsivity than proximal activity modulations. Intermittent theta burst stimulation (iTBS) increases vlPFC-dorsal striatal connectivity and striatal dopamine levels,^68,69^ which may help enhance goal-directed behaviours.^70^ Thus, we suggest that iTBS targeting the vlPFC-putamen network may help reduce habit bias and compulsivity in OCD.

Deficits in goal-directed behaviour are commonly observed within compulsive disorders but are less pronounced in non-compulsive disorders.^71^ As these deficits are more strongly associated with compulsivity than with a diagnosis of OCD,^22^ we propose that attenuated vlPFC-putamen connectivity, which underlies the shift away from goal-directed behaviour, could serve as a transdiagnostic marker for compulsive disorders.

While the current MB algorithm effectively assesses belief in task structure, it falls short of fully capturing disruptions in goal-directed behaviour due to its multidimensional nature. Compulsive checking is associated with difficulty recalling intentions for context-dependent planning.^72^ Therefore, the interplay between memory and learning needs to be incorporated into MB algorithms to understand how impaired prospective memory contributes to habit bias in compulsive disorders. Additionally, human goal-directed behaviour is influenced by the varying accuracy of internal models of task structure.^73^ When individuals form inaccurate models due to misconceptions, their goal-directed behaviour sometimes resembles complex model-free strategies. From this perspective, we should not overlook the possibility that incorrectly formed reasoning models may be related to habit bias in patients. Given the difference in beliefs (i.e. uncertainty) about model-based predictions but not in the prediction error itself, the present study partially supports that patients with OCD retain spared functions in their understanding of task structure.

This study has several limitations. First, our study included a moderate sample size, which may not be strong enough to ensure high replicability due to clinical complexity. To address this concern, we showed that our findings were consistently reproduced when tested under the population distribution estimated by the permutation tests and when controlling for the effects of medications and mood comorbidity. Permutation tests are robust to nuisance effects, as these tests involve repeatedly shuffling the group labels to create a null distribution. Second, although we tried to minimize the effects of cognitive behavioural therapy on goal-directed behaviours by recruiting patients who had not received this therapy in the previous year, long-term effects may remain.^74^

We suggest that the attenuated regulation of the habitual neural system via the vlPFC-putamen circuit underlies habit bias and compulsion in OCD. This finding indicates that the imbalance in goal-directed versus habitual actions is attributed to impaired top-down control by the prefrontal arbitrator, in addition to disruptions in each neural system. Network-level modulation enhancing the vlPFC-putamen circuit may be a viable neurotherapeutic approach for addressing compulsivity.

## Supplementary Material

awae102_Supplementary_Data

## Data Availability

Summary data used to generate the findings of this study will be available upon request to the lead contact. Individual participant data are available subject to participant consent. The analysis codes are available at github.com/takwan/arbitration-OCD.
