# Supplementary material for: Neurocomputational model of compulsivity: deviating from an uncertain goal-directed system
Source: Brain. 2024 Apr 8;147(6):2230–44. doi: 10.1093/brain/awae102 (PMC11146420; doi:10.1093/brain/awae102)
Supplement: awae102_Supplementary_Data [file awae102_supplementary_data.pdf]

Supplementary material

Neurocomputational model of compulsivity: deviating from an uncertain goal-directed system

Supplementary materials and methods

Participant inclusion and exclusion criteria

Patients who suffered moderate or more severe symptoms (Y-BOCS total ≥ 15) and had no history of cognitive behavioral therapy for at least a year were asked about their willingness to participate in this study. We recruited thirty-one HCs through internet advertisements and screened them using the SCID-I, non-patient edition.[1] Exclusion criteria were set as follows: a lifetime history of psychotic or neurological disorders, substance use disorder or mental retardation (intelligence quotient ≤ 70). Out of the original dataset of 66 subjects, 30 patients and 30 HCs remained after excluding four who withdrew their consent and two with incomplete datasets.

Computational model of arbitration

The model computed variables responsible for the arbitration process in the following computational framework.

Step 1: Model-based and model-free reinforcement learning. The learners updated action values for each state-action pair using prediction errors. An MB learner employed both FORWARD learning and BACKWARD planning, updating its action value (Q_{MB}) based on the SPE (δ_{SPE}), an error in the prediction of state-transition probability (T(s, a, s’)).[2]

$$\delta_{SPE} = 1 - T(s, a, s')$$

$$\Delta T(s, a, s') = \mu \delta_{SPE}$$

$$Q_{MB}(s, a) = \sum_{s'} T(s, a, s')\{r(s') + max_{a'} Q_{MB}(s', a')\},$$

where *a* and *a'* refer to the action in the current *s* and the next state *s'*, *r(s')* indicates the earned reward at *s'* state, and μ denotes the learning rate of the MB system. The MB agent additionally updated the action values for all actions using BACKWARD planning whenever an explicit goal was presented (i.e., change in a specific goal condition or transition from the universal to the specific goal condition).

$$r(s) = \begin{cases} R & for\ a\ goal\ state \\ 0 & otherwise \end{cases}$$

$for\ i = 3, 2$

$\quad for\ s\ \in S_{i-1}$

$\quad\quad Q_{MB}(s, a) = \sum_{s'} T(s, a, s')\{r(s_i) + max_{a'} Q_{MB}(s', a')\} \quad for\ all\ a,$

$\quad end$

end

where R is the reward corresponding to each goal state and S_i refers to the set of states in stage *i*.

On the other hand, an MF learner updated its action value (Q_{MF}) based on the RPE (δ_{RPE}) via the SARSA algorithm.[3] The temporal discount factor (γ) was fixed at 1 because the two-step decision task does not allow the effects of time delays on the perception of reward values.

$$\delta_{RPE} = r(s') + \gamma Q_{MF}(s', a') - Q_{MF}(s, a)$$

$$\Delta Q_{MF}(s, a) = \mu \delta_{RPE},$$

where μ refers to the learning rate of the MF system.

Step 2: Uncertainty estimation for model-based and model-free learning. The arbitrator estimated the uncertainty of each learning given the history of prediction errors. For MB learning, we used hierarchical Bayesian modeling to estimate the prediction uncertainty of the strategy given the SPE history.[4] The hierarchical form consisted of the following stages. First, we considered that the prediction error history (PE) has a likelihood given by parameters (θ_0, θ_1 and θ_2), each of which represents the probability of making zero, negative or positive prediction error.

$$P(PE|\theta) = \begin{cases} \theta_0 & if\ -\omega \leq PE \leq \omega \\ \theta_1 & if \qquad PE < -\omega \\ \theta_2 & if \qquad PE > \ \ \omega \end{cases}$$

where ω refers to the tolerance threshold determining whether the prediction error is zero, negative or positive. There was only the positive tolerance threshold for the MB system because SPE is always positive. The sum of θ_j equals 1. Second, we assumed that θ_j came from a Dirichlet distribution governed by a concentration hyperparameter λ_j (j = 0, 1 or 2).

$$(\theta_0, \theta_1, \theta_2) \sim Dirichlet(\lambda_0, \lambda_1, \lambda_2),$$

where the sum of the λ_j across the three prediction error cases equals 1. Then, we used conjugacy to estimate parameter θ_j of the posterior distribution:

$$Dirichlet(\lambda_0 + \#PE_0, \lambda_1 + \#PE_1, \lambda_2 + \#PE_2),$$

where $\#PE_j$ (j = 0, 1 or 2) refers to the number of events recognized to make the zero, negative or positive prediction error. The variance and mean of the posterior distribution were used to estimate the prediction uncertainty because its ratio (Fano factor) measures the dispersion or variability of a counting process.[5] Previous studies demonstrated that the inverse of the index could be used to capture the reliability of counting prediction errors.[4,6] We estimated the uncertainty of the MB strategy (χ_{MB}) using the Fano factor:

$$\pi_j = \frac{Var(\theta_j|D)}{E(\theta_j|D)}$$

$$\chi_{MB} = \pi_0 / \sum_{i=0}^{2} \pi_j,$$

where π_j refers to the uncertainty for each case of prediction leading to zero, negative or positive prediction error (j = 0, 1 or 2), D refers to a set of SPE history (i.e., total number of events that lead to zero, negative, and positive prediction errors), and *Var* and E denote the variance and expectation of the posterior $\theta_j|D$.

In contrast to the SPE, the range of RPE estimates varied across the goal states with different rewards. The hierarchical Bayesian method that applies the constant threshold of tolerance to the prediction error estimate across all trials would unstably determine the prediction error cases for MF learning. Alternatively, we used the Pearce-Hall associability learning theory to substitute for the Bayesian method.[4,7] This learning rule assumes that the associability of (or attention given to) an erroneous prediction becomes stronger following an unexpected consequence (e.g., lower or higher reward than expected) regardless of the sign of the prediction error.[7,8] As it uses the absolute value of the prediction error to modulate the associability, we made the MF agent update the absolute RPE estimate as follows:

$$\Delta\Omega = \eta(|RPE| - \Omega),$$

where Ω is the absolute RPE estimate and η refers to the learning rate of the model estimating Ω. In line with associability learning theory, attention is allocated to uncertain cues to acquire additional information for more accurate predictions in the future.[9,10] Thus, attention is considered an inference about the uncertainty of cues.[11] Based on the Pearce-Hall learning model, the uncertainty of MF learning (χ_{MF}) can be defined as follows:[4,6]

$$\chi_{MF} = \Omega / RPE_{max},$$

where the upper bound of RPE (RPE_{max}) is 40.

Step 3: Uncertainty-based competition between model-based and model-free learning. Following our previous study,[4] we implemented uncertainty-based competition between MB and MF learning by using a dynamic two-state transition model.[12] The transition rate (α) from

MF to MB learning was a function of χ_{MF}, while the rate of the opposite transition (β) was a function of χ_{MB}.

$$\alpha = A_\alpha/(1 + e^{B_\alpha(1-\chi_{MF})})$$

$$\beta = A_\beta/(1 + e^{B_\beta(1-\chi_{MB})}),$$

where A_α is the amplitude of the MF→MB transition rate function while A_β is that of the MB→MF transition rate function. Each B_α and B_β is the steepness of the corresponding transition rate function and is given as follows:

$$B_\alpha = \log(A_\alpha/\alpha_0 - 1)$$

$$B_\beta = \log\left(A_\beta/\beta_0 - 1\right),$$

where α_0 and β_0 are the boundary conditions determining the sensitivity of each transition rate function to the corresponding steepness. They were fixed at 0.01 via a model fit to an independent dataset.[4] Given the difference in the two transition rates (α and β), the arbitration model dynamically updated the probability of choosing the MB (P_{MB}) over the MF strategy at each action stage:

$$\frac{dP_{MB}}{dt} = \alpha(1 - P_{MB}) - \beta P_{MB}.$$

Step 4: Action selection based on the action value integrated from the two strategies. The action value (Q_{Arb}) of the arbitration system was computed by integrating the Q_{MB} and Q_{MF} in a weighted average manner by the control weight (i.e., P_{MB}) between the two individual systems:[2]

$$Q_{Arb}(s,a) = P_{MB}Q_{MB}(s,a) + (1 - P_{MB})Q_{MF}(s,a).$$

The arbitrator stochastically selected an action at each action stage via the softmax function of the Q_{Arb}:[2,13]

$$P(s,a) = \frac{e^{\tau \times Q_{Arb}(s,a)}}{\sum_b e^{\tau \times Q_{Arb}(s,b)}},$$

where $P(s,a)$ denotes the probability of a model agent's selection for a chosen action a given observed state-action pair and τ is the inverse softmax temperature. The decision parameter τ controlled the extent to which the agent made a choice with a higher action value. The higher τ is, the greater the difference in the selection probability for actions that differ in their action values.

Model fitting and evaluations

We fitted the model parameters to the observed behaviors for each individual by minimizing the negative log-likelihood of the model's selection probability for an action a chosen by a subject ($-\sum \log P(s,a)$), summed across trials. A local optimum was approximated using the Nelder-Mead simplex method with 1200 iterations for each initial parameter seed.[14] We chose the best optimized parameter set across 100 randomly generated initial parameter seeds.

We compared the goodness-of-fit (i.e., sum of the negative log-likelihood) of three different models explaining behavior on the decision task: one with the arbitration process between MB and MF systems and two without it (i.e., solely by MB or MF learning system). Two metrics that capture the goodness-of-fit while penalizing the complexity of a model were used for the model selection: Bayesian information criterion and Akaike information criterion. The Wilcoxon signed rank test was performed for the comparisons between the paired samples due to nonnormal distributions of the metrics.

A computational model should be identifiable to precisely infer human behavior (i.e., a unique value for each model parameter could be learned). To assess the identifiability of the arbitration model, we ran a parameter recovery analysis: 1) generating behavioral data using the model with the model parameters trained on the participants' actual behaviors (original parameters), 2) retraining the model using the same optimization method to fit the parameters

to the generated behaviors for each participant (recovered parameters), and 3) testing Pearson correlations between the original and recovered parameters.

To demonstrate how precisely the arbitration model captured variability in participants' choice behavior, we compared the probability that participants chose the left action in each state and the proportion of the left choice by the model in the same states. The participants' choice probability was split into 10 equal-sized bins across 0 to 100 percent, and the model's choice probability was averaged within each bin.

fMRI data acquisition and preprocessing

Using a Siemens Trio 3T MRI scanner with a 32-channel head coil, we scanned six sessions of task-based fMRI using an EPI sequence (repetition/echo time = 1,500/30 ms, flip angle = 85°, phase encoding direction = left-right, 2.3 mm isotropic voxels, 66 slices, multiband x3) for approximately 9 min per session while participants simultaneously performed the two-step decision task. To correct distortion in fMRI images, we acquired a pair of blip images with phase encoding directions in left-right and right-left. T1/T2-weighted MRI data were acquired using an MPRAGE sequence for the purpose of brain segmentation and coregistration. Each anatomical image was acquired with repetition/echo time = 2,400/2.19 or 3,200/565 ms, respectively, while sharing other imaging parameters (0.85 mm isotropic voxel dimension and 320 slices).

We preprocessed the neuroimaging data following the Human Connectome Project preprocessing pipeline.[15] We extracted non-brain tissues using a FNIRT-based masking method after aligning T1w and T2w images to the AC-PC line. To reduce magnetic susceptibility-induced distortion, the bias field of the structural images was corrected. To preprocess the functional images, we first realigned the fMRI time series for head motion correction using rigid body transformation to the first volume reference. The motion

parameters were provided for nuisance regression in first-level analysis following the preprocessing step. Because EPI images are susceptible to the magnetic field inhomogeneity effect in a phase encoding direction, we corrected the EPI distortion using the pair of blip images with reversed phase encoding directions in the FSL topup.[16] We coregistered the distortion-corrected functional images to the T1w images using the boundary-based registration method for fine tuning.[17] Subsequently, we performed nonlinear warping to register the functional images to the standard MNI space with a 2 mm isotropic voxel dimension. Finally, we applied spatial smoothing with a full width at half maximum Gaussian kernel of 6 mm to improve the signal-to-noise ratio.

Supplementary results

Demographic and clinical characteristics

Demographic variables were comparable between thirty patients and thirty HCs (Table 1). Patients suffered moderate severity of obsessive-compulsive symptoms (mean [SD] $\text{Y-BOCS}_{\text{Obsession}}$ = 11.50 [3.26] and mean [SD] $\text{Y-BOCS}_{\text{Compulsion}}$ = 10.60 [2.92]) and had no history of cognitive behavioral therapy for at least a year.

Parameter recoverability of the computational model

As demonstrated in previous studies,[4,6] the parameter recovery analysis showed significant correlations between the original and recovered parameters estimated using the arbitration model: the threshold for defining zero SPE (r = 0.73, P < 0.001), the learning rate of the model estimating absolute RPE (r = 0.69, P < 0.001), the amplitude of the MB$\rightarrow$MF transition rate function (r = 0.74, P < 0.001), the amplitude of the MF$\rightarrow$MB transition rate

function (r = 0.73, $P < 0.001$), the inverse softmax temperature (r = 0.45, $P < 0.001$), and the learning rate of MB and MF systems (r = 0.42, $P < 0.001$).

Differences of the model parameters between patients and HCs

Regarding the arbitration model, the inverse softmax temperature was lower in patients compared to HCs (t = -3.01, P_{FWER} = 0.024). Other model parameters were comparable between groups: the threshold for the threshold for defining zero SPE (P_{FWER} = 0.078), the learning rate of the model estimating absolute RPE (P_{FWER} = 0.57), the amplitude of a transition rate function from MB to MF learning (P_{FWER} = 0.084), the amplitude of a transition rate function from MF to MB learning (P_{FWER} = 0.544), and the learning rate of MB and MF systems (P_{FWER} = 0.672).

Supplementary Table 1. Comparisons of the goodness-of-fit among computational models

Metric	Arbitration[a]		MB alone[b]		MF alone[b]		Arbitration vs. MB		Arbitration vs. MF	
	**Median**	**MAD**	**Median**	**MAD**	**Median**	**MAD**	***z***	***P***	***z***	***P***
BIC	513.1	100.6	548.0	94.3	821.2	93.0	-4.22	< 0.001	-6.37	< 0.001
AIC	487.9	100.8	523.0	94.0	812.9	93.0	-4.22	< 0.001	-6.54	< 0.001

Abbreviations: BIC, Bayesian information criterion; AIC, Akaike information criterion
[a] model parameters of the arbitration model: the threshold for defining zero SPE (ω), the learning rate of the model estimating absolute RPE (η), the amplitude of a transition rate function from MB to MF learning (A_β), the amplitude of a transition rate function from MF to MB learning (A_α), inverse softmax temperature (τ), and the learning rate of MB and MF systems (μ)
[b] model parameters included in the MB or MF alone model: the inverse softmax temperature and the learning rate of each system

Supplementary Table 2. Brain activation and connectivity during the arbitration process between goal-directed and habitual decision-making

Brain region	MNI coordinate (mm)	Cluster size (k_E)	One-sample *t*-test (*N* = 60)		
			t^a	*df*	Cluster P_{FWER}
			Main effect GLM 2. Prediction error estimation		
**State-Prediction Error**					
R dlPFC	46, 32, 22	775	8.3	59	< 0.001
L Insula	-34, 18, -2	140	8.3	59	< 0.001
R SFG medial	2, 32, 46	314	7.5	59	< 0.001
L dlPFC	-48, 26, 28	608	7.3	59	< 0.001
R Insula	40, 22, 0	139	6.8	59	< 0.001
**Reward-Prediction Error**					
L NAcc	-6, 6, -8	104	7.7	59	< 0.001
R NAcc	16, 12, -10	84	6.4	59	< 0.001
L Putamen[b]	-30, -14, 8	72	4.3	59	0.002
R Putamen[b]	26, -16, 2	20	3.5	59	0.016
			Main effect GLM 3. Uncertainty estimation		
**Uncertainty of MB learning (χ_{MB})**					
L Insula/IFG	-38, 18, -4	946	-11.0	59	< 0.001
R IFG	50, 18, 8	3236	-8.9	59	< 0.001
**Uncertainty of MF learning (χ_{MF})**					
L Insula/IFG	-28, 20, -10	990	-10.9	59	< 0.001
R IFG	44, 24, 4	3431	-9.6	59	< 0.001
**Maximum of the uncertainty of MB and MF strategies, max(χ_{MB}, χ_{MF})**					
R IFG	50, 18, 8	3953	-8.4	59	< 0.001
L IFG	-50, 20, 6	747	-4.8	59	< 0.001
			Main effect GLM 4. Value estimation		
**Chosen action value of MB learning (Q_{MB})**					
R Premotor cortex	40, -16, 52	915	7.5	59	< 0.001
L SFG medial	-6, 60, 12	1857	5.3	59	< 0.001
**Chosen action value of MF learning (Q_{MF})**					
R SMA	8, 4, 54	72	6.0	59	< 0.001
L Putamen[b]	-22, 2, 2	49	4.1	59	0.012
**Value difference of arbitration system (chosen minus unchosen Q_{Arb})**					
R IPS	26, -48, 42	360	7.4	59	< 0.001
R Cerebellar crus II	-6, -86, -30	878	5.9	59	< 0.001
L IPS	-34, -60, 58	301	5.6	59	< 0.001
			Psychophysiological interaction GLMs		
**Connectivity of L IFG[c] when choosing MB over MF strategy (× P_{MB})**					
ns	–	–	–	–	–

**Connectivity of R IFG[c] when choosing MB over MF strategy (× P_{MB})**					
R NAcc	10, 10, -10	120	-5.1	59	0.009
R Putamen	32, -12, -4	73	-4.8	59	0.008
L Premotor cortex	-30, -2, -60	366	5.9	59	< 0.001
R Premotor cortex	32, 0, 58	388	5.3	59	< 0.001
**Connectivity of L putamen[d] when choosing MB over MF strategy (× P_{MB})**					
R IFG	60, 24, 18	130	-4.8	59	0.003
**Connectivity of R putamen[d] when choosing MB over MF strategy (× P_{MB})**					
L IFG	-52, 24, -2	601	-5.6	59	< 0.001
R IFG	56, 24, 22	973	-5.6	59	< 0.001
R TPJ	64, -46, 10	1161	-5.3	59	< 0.001
L Cerebellar lobule VIII	-28, -66, -54	211	-4.8	59	< 0.001
R IPS	56, -40, 48	192	-4.3	59	< 0.001
L TPJ	-54, -48, 22	446	-4.0	59	< 0.001

Abbreviations: dlPFC, dorsolateral prefrontal cortex; IFG, inferior frontal gyrus; IPS, intraparietal sulcus; NAcc, nucleus accumbens; SFG, superior frontal gyrus; SMA, supplementary motor area; TPJ, temporoparietal junction

[a] Positive/negative *t* value indicates activation/deactivation or positive/negative connectivity

[b] Small volume correction (10 mm radius sphere) on the left/right putamen [±27, -13, 4] implicated in MF learning[18]

[c] First eigenvariate of the left/right IFG signal (from a 5 mm radius sphere on MNI [-50, 20, -6] or MNI [50, 18, 8]) found to encode the maximum uncertainty of MB and MF strategies

[d] First eigenvariate of the left/right putamen signal (from a 5 mm radius sphere on MNI [±27, -13, 4]) implicated in MF learning[18]

Supplementary Table 3. Post-hoc permutation test results of the group comparisons of brain activation and connectivity

Brain region	MNI coordinate (mm)	Cluster size (k_E)	OCD vs. HCs	
			Pseudo *t*[a]	Corrected *P*
**Uncertainty of MB learning (χ_{MB})**				
R IFG	52, 24, 26	293	-3.9	0.015
**Value difference of arbitration system (chosen minus unchosen Q_{Arb})**				
R IPS	32, -46, 40	284	5.1	0.015
**Connectivity of R putamen[b] when choosing MB over MF strategy (× P_{MB})**				
L IFG	-46, 8, 18	174	-5.2	0.017
R IFG	60, 22, 20	34	-4.4	0.017
R IPS	54, -42, 34	33	-4.3	0.017

[a] Positive/negative *t* value indicates activation/deactivation or positive/negative connectivity
[b] First eigenvariate of the right putamen signal (from a 5 mm radius sphere on MNI [27, -13, 4]) implicated in MF learning[18]

References

1. First MB, Spitzer RL, Gibbon M, Williams JBW. *Structured clinical interview for DSM-IV-TR axis I disorders, research version, non-patient edition*. New York, NY: Biometrics Research, New York State Psychiatric Institute; 2002.

2. Glascher J, Daw N, Dayan P, O'Doherty JP. States versus rewards: dissociable neural prediction error signals underlying model-based and model-free reinforcement learning. *Neuron* 2010;66(4):585-595.

3. Sutton RS, Barto AG. *Reinforcement learning: an introduction*. Cambridge, MA: MIT Press; 1998.

4. Lee SW, Shimojo S, O'Doherty JP. Neural computations underlying arbitration between model-based and model-free learning. *Neuron* 2014;81(3):687-699.

5. Rajdl K, Lansky P, Kostal L. Fano Factor: a potentially useful information. *Front Comput Neurosci* 2020;14:569049.

6. Kim D, Park GY, JP OD, Lee SW. Task complexity interacts with state-space uncertainty in the arbitration between model-based and model-free learning. *Nat Commun* 2019;10(5738):1-14.

7. Pearce JM, Hall G. A model for pavlovian learning: variations in the effectiveness of conditioned but not of unconditioned stimuli. *Psychol Rev* 1980;87(6):532-552.

8. Roesch MR, Esber GR, Li J, Daw ND, Schoenbaum G. Surprise! neural correlates of Pearce-Hall and Rescorla-Wagner coexist within the brain. *Eur J Neurosci* 2012;35(7):1190-1200.

9. Hogarth L, Dickinson A, Austin A, Brown C, Duka T. Attention and expectation in human predictive learning: the role of uncertainty. *Q J Exp Psychol* 2008;61(11):1658-1668.

10. Beesley T, Nguyen KP, Pearson D, Le Pelley ME. Uncertainty and predictiveness determine attention to cues during human associative learning. *Q J Exp Psychol* 2015;68(11):2175-2199.

11. Feldman H, Friston KJ. Attention, uncertainty, and free-energy. *Front Hum Neurosci* 2010;4:215.

12. Dayan P, Abbott LF. *Theoretical neuroscience: computational and mathematical modeling of neural systems*. Cambridge, MA: MIT Press; 2001.

13. Luce RD. *Individual choice behavior: a theoretical analysis*. Mineola, NY: Dover Publication; 1959.

14. Lagarias JC, Reeds JA, Wright MH, Wright PE. Convergence properties of the Nelder-Mead simplex method in low dimensions. *SIAM J Optim* 1998;9(1):112-147.

15. Glasser MF, Sotiropoulos SN, Wilson JA, *et al*. The minimal preprocessing pipelines for the Human Connectome Project. *NeuroImage* 2013;80:105-124.

16. Andersson JL, Skare S, Ashburner J. How to correct susceptibility distortions in spin-echo echo-planar images: application to diffusion tensor imaging. *NeuroImage* 2003;20(2):870-888.

17. Greve DN, Fischl B. Accurate and robust brain image alignment using boundary-based registration. *NeuroImage* 2009;48(1):63-72.

18. Wunderlich K, Dayan P, Dolan RJ. Mapping value based planning and extensively trained choice in the human brain. *Nat Neurosci* 2012;15(5):786-791.
